# Electronic media use and sleep in children and adolescents in western countries: a systematic review

**DOI:** 10.1186/s12889-021-11640-9

**Published:** 2021-09-30

**Authors:** Lisbeth Lund, Ida Nielsen Sølvhøj, Dina Danielsen, Susan Andersen

**Affiliations:** National Institute of Public Health, University of Southern Denmark, Studiestræde 6, DK-1455 Copenhagen, Denmark

**Keywords:** Systematic review, Child, Adolescent, Telecommunications, Sleep

## Abstract

**Background:**

Sleep is essential for child and adolescent health and well-being. There is an increasing interest in whether electronic media use affects children and young adolescents’ sleep. Prior reviews have focused on a school-aged population. Moreover, it is crucial that research continuously addresses the processes of technology and media use and the implication on sleep. This systematic review examines the evidence of electronic media use related to sleep among 0–15-year-olds.

**Methods:**

Searches were carried out in four databases (CINAHL, Web of Science, EMBASE, and Medline). Inclusion criteria included age ≤ 15 years, and intervention, cohort, or cross-sectional studies from western countries. Methodological quality was rated using the Quality Assessment Tool for Quantitative Studies by two independent reviewers. Data was extracted using a standardized data extraction form. Synthesis was done by summarizing results across studies by age groups of 0–5, 6–12, and 13–15 years within four sleep domains: Bedtime and sleep onset; Sleep quality; Sleep duration; Daytime tiredness.

**Results:**

The search identified 10,719 unique studies, of which 109 fulfilled inclusion and exclusion criteria and were assessed for methodological quality. In total, 49 studies were included in the review. The study designs were randomized controlled trials (*n* = 3), quasi-experimental studies (*n* = 2), prospective cohort studies (*n* = 15), and cross-sectional studies (*n* = 29). Evidence for an association between electronic media use and sleep duration was identified, with stronger evidence for 6–15-years-olds than 0–5-year-olds. The evidence for a relationship between electronic media use and other sleep outcomes was more inconclusive. However, for 6–12-year-old children, there was evidence for associations of electronic media use with delayed bedtime and poor sleep quality. For 13–15-year-olds, there was evidence for associations between screen time and problems falling asleep, and between social media use and poor sleep quality.

**Conclusions:**

Overall, electronic media use was generally associated with shorter sleep duration in children and adolescents. Studies with stronger research design and of higher quality are needed to draw solid conclusions about electronic media’s impact on other sleep outcomes. Public awareness and interventions could be promoted about the potential negative impact on children’s sleep of electronic media devices that are used excessively and close to bedtime.

**Supplementary Information:**

The online version contains supplementary material available at 10.1186/s12889-021-11640-9.

## Background

Sleep has a major impact on the health and well-being of children and adolescents. Sleep is vital for development and learning ability, and insufficient sleep over an extended period can have long-term physical and psychological health implications [1]. Physiological and psychological changes that emerge in childhood and youth may impact negatively on sleep, but poor sleep is arguably also related to, or compounded by, external factors such as early school start times, environmental conditions in the bedroom (e.g. noise, high temperature or too much light) and the availability of electronic media [1–3]. *Over the last few decades, a major lifestyle change has happened due to the incorporation of electronic media device use into people’s daily life. Electronic media has become a core part of young people’s lives and children today are growing up surrounded by electronic media devices.* Studies demonstrate that most children, even as young as four months of age, have experience with using electronic media devices, although electronic media consumption is largest among older teenagers [4]. M*edia devices have become an integral part of children’s development environment and a prevalent mode of communication among adolescents* [5]*. Although electronic media is widely accepted and accessible in the home environment, there is yet only a limited understanding of how access to and use of electronic media may impact the sleep patterns of children and adolescents.*

Technology is continuously evolving, and the way electronic media devices are used in everyday life may change over time. Although previous systematic reviews and meta-analyses have established a correlation between media use and sleep [6–8], it is crucial that research continuously addresses the processes of technology and media use and its implication on children and adolescents’ sleep patterns. Therefore, reviews that include the newest types of electronic media devices and technological trends are needed. Moreover, previous reviews on this subject focus mainly on an older paediatric target group, and there is limited knowledge about the evidence of electronic media devices and the impact on sleep among pre-schoolers [9].

The aim of this study was to systematically review the literature on the impact of using electronic media on sleep in children and adolescents. The population was pre-school children, school-age children up to 12-years-old and young adolescents up to 15 years old. The exposure was access to and use of electronic media devices, and the outcomes were bedtime and sleep onset, sleep quality during nighttime, sleep duration and daytime tiredness. The intention was to inform policy and practice and to highlight what further research is needed on this topic.

## Methods

*A narrative method was applied to synthesis the data on the association between electronic media use and sleep among children and adolescents.*

### Eligibility criteria

We included studies which fulfilled the following eligibility criteria: (1) Assessed the associations between the use of or access to electronic media devices and sleep, i.e. delayed bedtime, sleep onset latency, sleep quality during night-time, sleep duration and daytime tiredness; (2) Published in English between January 1, 2009 and August 31, 2019; (3) From western countries; (4) Examined children and adolescents between 0 and 15.9 years of age without any diagnoses/diseases. Electronic media devices were defined as mobile phones, televisions, touchscreens/tablets, computers, or video game consoles. The exclusion criteria were apps intended to treat sleep disorders, or problems (e.g., sleep apnoea), and studies examining electromagnetic radiation.

### Data sources and search strategy

The PICo model was used to generate the search strategy, and the search strategy was divided into three search blocks: P (Patient / Problem / Population), I (Phenomenon of interest), and Co (Context). The population was children and adolescents aged 0–15; the field of interest was electronic media devices, and the context was before sleep (bedtime and sleep onset), during sleep (sleep quality during nighttime), and after sleep (sleep duration and daytime tiredness). We performed a systematic search in four databases: CINAHL, EMBASE, Web of Science, and Medline based on keywords (subject headings/MeSH terms) and free text searches (title, keywords, text). The search terms and syntax included relevant synonyms for the search terms adolescents/children (e.g. minor, teenager), electronic media devices (e.g. cell phone, screen), and sleep (e.g. sleep latency, bedtime routine) (see Supplementary eTable 1 for the full search in each database). In addition, we included previous reviews to identify relevant studies.

### Study selection; screening, quality assessment, and data extraction

Title and abstracts identified were screened for eligibility, and full texts of potentially eligible articles were read and assessed by two reviewers (LL and INS) independently. Discordance regarding inclusion was resolved through discussion. Two reviewers (LL and SA) independently assessed the methodological quality of the included quantitative studies. To ensure consistency in the quality assessments, meetings were held on an ongoing basis, focusing on inter-rater reliability. The methodological quality of the quantitative articles was assessed using the Effective Public Health Practice Project (EPHPP) assessment tool [10], based on the following five components: selection bias, study design, confounders, data collection methods, and withdrawal/dropouts. The EPHPP covers any quantitative study design, it is developed for use within public health and has been found to have psychometric properties as good as the Cochrane risk-of-bias tool. Each study was rated as high, moderate, or low quality. Low-quality studies were excluded to ensure moderate evidence. We extracted the data using a standardized data extraction form. It included country of study, age, sex, study design, sleep outcomes, exposure (electronic media device measures) and reported associations. Synthesis was done by summarizing results and conclusions across studies grouped by age groups of 0–5.9 years, 6–12.9 years, and 13–15.9 years within four sleep domains: Bedtime and sleep onset; Sleep quality; Sleep duration; Daytime tiredness. If a study included data on ages overlapping the defined age categories, we used the mean age to allocate the study to an age category. A few studies (*n* = 2) included a large age span. However, these studies had performed analyses by subgroups of age corresponding to our categories.

## Results

**Fig. 1:**
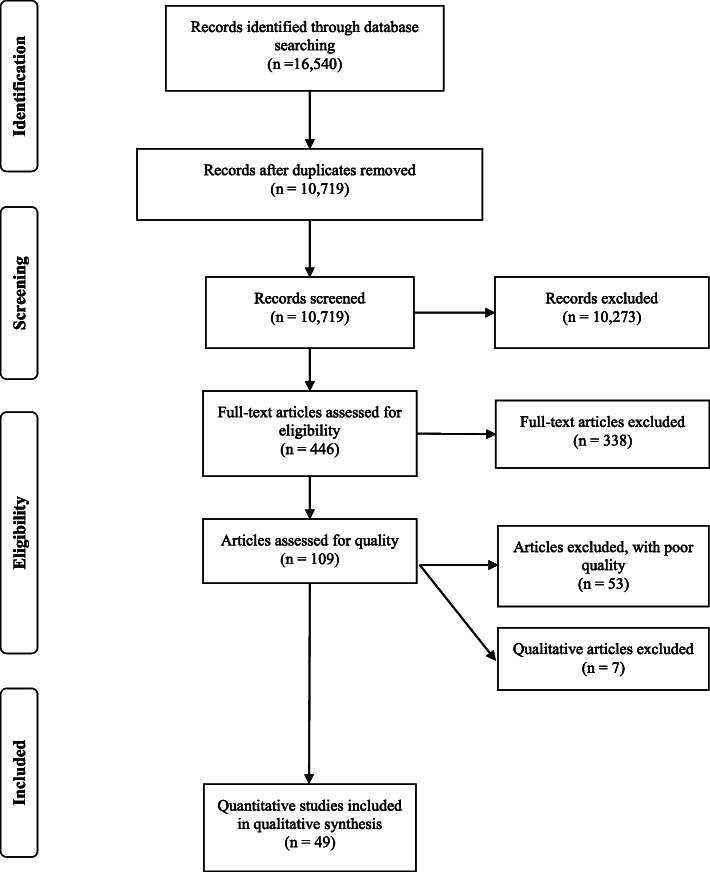
Flow chart of the review process

A total of 446 full texts were reviewed, of which 338 were excluded (Fig. 1). We identified 7 qualitative studies of which 3 studies received high- or moderate-quality assessments. These studies focused on factors (e.g. the role of parents) that facilitate the accessibility and acceptability of electronic media use in relation to sleep*,* and not on how electronic media use might impact sleep in children and adolescents. The results from the qualitative studies will be reported in a separate paper given the focus on quantitative studies in this systematic review.. In total, 52 quantitative studies were not included because they received a low-quality assessment rating as a result of a range of methodological issues: weakness in study design, a small percentage of responses, not controlling for confounders, and not reporting validity and reliability of measures used. Of the 49 included quantitative studies, four studies received a high-quality assessment rating, and 45 studies received a moderate-quality assessment rating. Of the included studies, 18 were conducted in North America (USA and Canada) [11–28], 23 in Europe (England, Finland, Sweden, Holland, Switzerland, France, Italy, Spain, and Germany) [29–51], five in Australia and New Zealand [52–56], and three studies combined several western countries [57–59]. There were three randomized controlled trials (RCTs) [27, 28, 50], two quasi-experimental studies [26, 51], 15 prospective cohort studies [12, 16, 19, 24, 30, 32, 35, 37, 40, 42, 45, 47, 48, 52, 55], and 29 cross-sectional studies [11, 13–15, 17, 18, 20–23, 25, 29, 31, 33, 34, 36, 38, 39, 41, 43, 44, 46, 49, 53, 54, 56–59] (see Table 1 for chacteristics of the included studies)*. The majority of the included studies adjusted for all or most of the following confounders: ethnicity, age/grade, sex, and socioeconomic status (*e.g.*, parental education and/or parental occupation). Some studies also included health factors such as BMI, psychological symptoms, and physical activities.* Detailed descriptions of study design and results are available in Supplementary eTables 2–4, and references on excluded studies in Supplementary eTable 5.

**Table 1:** Study characteristics of the 49 included studies in the systematic review

	n (%)
**Geographic location**	
North America	18 (37)
Europe	23 (47)
Australia and New Zealand	5 (10)
Several western countries included	3 (6)
**Age range**	
0–5 years	11 (22)
6–12 years	13 (27)
13–15 years	23 (47)
0–15 years^a^	2 (4)
**Study design**	
RCT study	3 (6)
Quasi-experimental study	2 (4)
Prospective cohort study	15 (31)
Cross-sectional study	29 (59)
**Electronic media devices**^b^	
Television	18 (37)
Video game	12 (25)
Mobile phone/smartphone	13 (27)
Computer/internet	14 (29)
Touchscreen/tablet	3 (6)
Social media	7 (14)
Total screen time	43 (21)
Electronic media devices in the bedroom	5 (10)
Media content (e.g., violent content)	1 (2)
**Sleep outcome**^**c**^	
Delayed bedtime/sleep onset latency	22 (45)
Poor sleep quality	19 (29)
Short sleep duration	36 (73)
Daytime tiredness	15 (31)

^a^We have predefined three age group categories. If a study included data on ages overlapping the defined age categories, we used the mean age to allocate the study to an age category. A few studies (n = 2) included a large age span

^b^As some articles examine several electronic media devices within the same study, this column does not add up to 100%

^c^As some articles examine several sleep outcomes within the same study, this column does not add up to 100%

### Electronic media use among children aged 0–5

Table 2 summarizes the results of the 13 studies [11–15, 27, 29–32, 52, 56, 57] included for 0–5-year-olds.

**Table 2:** Summary of studies and their findings on the relationship between electronic media devices and sleep outcomes among 0–5-year-olds

Electronic media device	Measured at bedtime	Sleep outcomes				Study design	Quality Assessment
		Delayed bedtime or sleep onset latency	Poor sleep quality	Short sleep duration	Daytime sleep duration/ Daytime tiredness		
**Television**							
Beyens 2019	No	+		+/0	+	Cross-sectional	Moderate
	Yes	+		+/0	0		
Cespedes 2014	No			+		Cohort study	Moderate
Cheung 2017	No	0	0	0	+	Cross-sectional	Moderate
Marinelli 2014	No			+		Cohort study	Moderate
McDonald 2014	Yes			+		Cross-sectional	Moderate
Moorman 2019	No	0		+	+	Cross-sectional	Moderate
	Yes	+		+	0		
Plancoulaine 2018	No			+		Cohort study	Moderate
**Video game (console)**							
Beyens 2019	No	0		0	0	Cross-sectional	Moderate
	Yes	0		0	0		
Moorman 2019	No	+		0	0	Cross-sectional	Moderate
	Yes	+		0			
Nathanson 2018	No	0		0	0	Cross-sectional	Moderate
	Yes	0		0	0		
**Mobile phone**							
Beyens 2019	No	0		0	+/0	Cross-sectional	Moderate
	Yes	0		0	+		
Nathanson 2018	No	0		0	0	Cross-sectional	Moderate
	Yes	+		0	0		
**Touchscreen/tablet**							
Beyens 2019	No	+		+/0	+	Cross-sectional	Moderate
	Yes	+		+/0	+/0		
Cheung 2017	No	+	0	+	0	Cross-sectional	Moderate
Nathanson 2018	No	+		+	0	Cross-sectional	Moderate
	Yes	+		+	0		
**Computer/internet**							
Beyens 2019	No	0		0	0	Cross-sectional	Moderate
	Yes	0		0	0		
Moorman 2019	No	0		+/0	0	Cross-sectional	Moderate
	Yes	0		0	0		
Nathanson 2018	No	0		0	0	Cross-sectional	Moderate
	Yes	0		0	0		
**Total screen time**							
Parent 2016	No		+	+		Cross-sectional	Moderate
Ribner 2019	No			+	0	Cross-sectional	Moderate
Xu 2016	No	+	–	+		Cohort study	Moderate
Zhang 2018	No		0	0		Cross-sectional	Moderate
**Electronic media devices in the bedroom**							
Cespedes 2014				+/0		Cohort study	Moderate
Moorman 2019		+		0	0	Cross-sectional	Moderate
**Media content**							
Garrison 2012	No		+			RCT	Moderate

Note: 1st author and year. (+) Significant positive association; (0) No association; (−) Significant negative association; (+/0) refers to both a positive association and no association in different subgroups

#### Bedtime and sleep onset

Five studies analysed the association between electronic media use and late bedtime and/or sleep onset [11, 13, 14, 29, 52]. Three studies examined tablet use and found an association of tablet use (both general and at bedtime) with delayed bedtime or sleep onset latency [11, 13, 14]. There were two studies on television viewing before bedtime and both found an association with delayed bedtime or sleep onset latency [11, 13]. Two studies estimated the association between mobile phone use and delayed bedtime or sleep onset latency with one finding a positive association for the use of mobile phones at night-time [14] and one finding no association [11]. The association between gaming and delayed bedtime or sleep onset latency was examined in three studies [11, 13, 14] with one study reporting an association [13]. One longitudinal study examined screen time in 2-year-olds and found a positive association with sleep onset latency in 5-year-olds [52]. No evidence was found for an association between computer/Internet use and delayed bedtime/sleep onset [11, 13, 14]. One study found an association between the presence of a television in the bedroom and delayed bedtimes on weekdays, but not on weekends [13].

#### Sleep quality

There was no evidence for television viewing or use of touchscreens [29], while there was inconsistent evidence for general screen time use [15, 52, 56] on night awakenings or sleep disturbances. An intervention study showed that promotion of prosocial content on electronic media reduced sleep problems [27].

#### Sleep duration

Regarding sleep duration, three studies showed that overall screen time was associated with shorter sleep duration [15, 52, 57], and six studies showed this concerning television viewing [11–13, 30–32] and three studies concerning use of touch screen or tablet [11, 14, 29]. Lack of association was found for the use of mobile phones [11, 14], video gaming [11, 13, 14] and computers or Internet [11, 13, 14]. Inconsistent evidence was found for the presence of electronic media devices in the bedroom [12, 13]; for ethnic minority children, television in the bedroom among 4-year-olds was associated with 32 fewer minutes of sleep per day at age 7 [12].

#### Daytime sleep duration

Three studies found associations between television viewing and longer naps [11, 13, 29], while inconsistent evidence was found for the use of mobile phones [11, 14] and touch screen or tablet [11, 14, 29]. No evidence was found for gaming, computer or internet use, and the presence of electronic media devices in the bedroom [11, 13, 14].

### Electronic media use among children aged 6–12

Table 3 summarizes the results for the 15 studies [15–21, 28, 30, 33–37, 53] included for the age range 6–12.

**Table 3:** Summary of studies and their findings on the relationship between electronic media devices and sleep outcomes among 6–12-year-olds

Electronic media device	Measured at bedtime	Sleep outcomes				Study design	Quality Assessment
		Delayed bedtime or sleep onset latency	Poor sleep quality	Short sleep duration	Daytime tiredness		
**Television**							
Arora 2014	Yes	0	+	+		Cross-sectional	Moderate
Chahal 2012	Yes			+		Cohort study	Moderate
Falbe 2014	No	+		+		Cross-sectional	Moderate
Marinelli 2014	No			+		Cohort study	Moderate
Mireku 2019	Yes	+	+	+		Cross-sectional	Moderate
Nuutinen 2013	No	+		+		Cohort study	Moderate
Yland 2015	No			+		Cross-sectional	Moderate
**Video game (console)**							
Arora 2014	Yes	+	+	+		Cross-sectional	Moderate
Chahal 2012	Yes			+		Cohort study	Moderate
Falbe 2014	No	+		+		Cross-sectional	Moderate
Yland 2015	No			0		Cross-sectional	Moderate
**Mobile phone**							
Arora 2014	Yes	+/0	+	+		Cross-sectional	Moderate
Chahal 2012	Yes			+		Cohort study	Moderate
Huss 2015	No	+	+	+	0	Cohort study	Moderate
Mireku 2019	Yes	+	+	+		Cross-sectional	Moderate
Redmayne 2013	No	0	0	0	+	Cross-sectional	Moderate
**Computer**							
Arora 2014	Yes	+/0	+	+		Cross-sectional	Moderate
Chahal 2012	Yes			+		Cohort study	Moderate
Nuutinen 2013	No	+		+		Cohort study	Moderate
Yland 2015	No			+/0		Cross-sectional	Moderate
**Internet/social media**							
Arora 2014	Yes	+	0	+		Cross-sectional	Moderate
**Total screen time**							
Barlett 2011	No			+		Cohort study	Moderate
Brambilla 2017	Yes			+		Cross-sectional	Moderate
Gentile 2014	No			+		Cohort study	Moderate
Greever 2017	No		+	0		Cross-sectional	Moderate
Mireku 2019	Yes	+		+		Cross-sectional	Moderate
Parent 2016	No		+	+		Cross-sectional	Moderate
**Electronic media devices in the bedroom**							
Arora 2014		0	0	+		Cross-sectional	Moderate
Brambilla 2017				0		Cross-sectional	Moderate
Chahal 2012				+		Cohort study	Moderate
Falbe 2014		+		+		Cross-sectional	Moderate
Mindell 2016				0		RCT	Moderate
Nuutinen 2013		+		+/0		Cohort study	Moderate

Note: 1st author and year. (+) Significant positive association; (0) No association; (+/0) refers to a positive association and no association for different outcome measures

#### Bedtime and sleep onset

Six studies analysed the association between electronic media use and late bedtime and/or sleep onset [18, 33, 35–37, 53]. Five of the studies found an association. Some studies only found an association when stratified by specific variables such as weekends/weekdays. For example, Mireku et al. (2019) showed that use of screen-based media device in the last hour before bedtime was associated with 1.44 times the odds of delayed sleep onset on weekends, but no association was found on weekdays [36]. Two studies assessed the association between video gaming and sleep onset [18, 33]. Arora et al. (2014) showed that high frequency of gaming at bedtime was associated with a 6.2 min prolonged sleep onset on weekdays [33]. Falbe et al. (2015) reported that each hour per day of gaming was associated with a 9.8 min later bedtime [18]. Two studies found that electronic media in the bedroom, including mobile phones, televisions, and computers, were associated with later bedtimes [18, 37]. One of the studies, however, only found a significant association among boys, not girls [37]. One study did not find that electronic media devices in the bedroom was associated with sleep latency and trouble falling asleep [33].

#### Sleep quality

Six of eight studies found a positive association between the use of electronic media, including total screen time and bedtime use of television and mobile phone, and night-time awakenings/sleep disturbances [15, 33, 35, 53] or poor sleep quality [20, 36]. For example, Mireku et al. (2019) found that using mobile phone or watching television in the dark was associated with restless sleep, waking up at night, and waking early in the morning [36].

#### Sleep duration

A total of 15 studies were identified, examining the association between electronic media use and sleep duration among 6–12-year-olds [15–21, 28, 30, 33–37, 53]. The studies found use of mobile phone [17, 33, 35, 36], social media [33], and computer or television [17, 18, 21, 30, 33, 36, 37] associated with short sleep duration. Six studies examined the association between electronic media in the bedroom and sleep duration [17, 18, 28, 33, 34, 37]. Among these studies, three found an association. Chahal et al. (2012) showed a dose-response association, where children who had access to more electronic media in their bedroom slept less [17]. Falbe et al. (2015) found that children who slept close to a small screen (e.g., mobile phone) reported 21 min less sleep compared to children who did not [18].

#### Daytime tiredness

Two studies examined mobile phone use and daytime tiredness [35, 53]. Redmayne et al. (2013) found that children disturbed by their mobile phone at night at least once a week were 3.5 times more likely to experience daytime tiredness than children who were not disturbed by their mobile phone at night [53]. Huss et al. (2015) did not find an association [35].

### Electronic media use among children aged 13–15

Table 4 summarizes the results of the 24 studies [15, 22–26, 38–51, 54, 55, 58, 59] included in the age range 13–15.

**Table 4:** Summary of studies and their findings on the relationship between electronic media devices and sleep outcomes among 13–15-year-olds

Electronic media device	Measured at bedtime	Sleep outcomes				Study design	Quality Assessment
		Delayed bedtime or sleep onset latency	Poor sleep quality	Short sleep duration	Daytime tiredness		
**Television**							
Arora 2013	Yes			+		Cross-sectional	Moderate
Brunetti 2016	No			–	0	Cross-sectional	Moderate
Lange 2015	No		0			Cross-sectional	Moderate
Poulain 2019	No	0	0		0	Cohort study	High
Tavenier 2017	No	0		0		Cohort study	High
Twenge 2017	No			+/0		Cross-sectional	Moderate
**Video game (console)**							
Arora 2013	Yes			+		Cross-sectional	Moderate
Brunetti 2016	No			+/0	0	Cross-sectional	Moderate
Lange 2015	No		+/0			Cross-sectional	Moderate
Tavernier 2017	No	0		0		Cohort study	High
Wallenius 2009	No	+/−			+	Cross-sectional	Moderate
**Mobile phone/smartphone**							
Arora 2013	Yes			+		Cross-sectional	Moderate
Brunetti 2016	No			+	+	Cross-sectional	Moderate
Foerster 2019	No	+	+/0		0	Cohort study	Moderate
Lange 2015	No		0			Cross-sectional	Moderate
Poulain 2019	No	0	0		0	Cohort study	High
Tavernier 2017	No	0		+		Cohort study	High
**Computer/internet**							
Arora 2013	Yes			+		Cross-sectional	Moderate
Brunetti 2016	No			+	+	Cross-sectional	Moderate
Lange 2015	No		+/0			Cross-sectional	Moderate
Nuutinen 2014	No			+		Cross-sectional	Moderate
Poulain 2019	No	+	0		+	Cohort study	High
Tavernier 2017	No	0		+		Cohort study	High
Twenge 2017	No			+		Cross-sectional	Moderate
**Social media**							
Scott 2019	No	+	+			Cross-sectional	Moderate
Tavernier 2017	No	0		0		Cohort study	High
Twenge 2017	No			+		Cross-sectional	Moderate
Van der Schuur 2019	No	+			+/0	Cohort study	Moderate
Vernon 2015	No		+			Cross-sectional	Moderate
Vernon 2017	No		+			Cohort study	Moderate
**Total screen time**							
Bickham 2018	No			+		Quasi-experimental	High
Calamaro 2009	Yes	+		+	+	Cross-sectional	Moderate
Das_Friebel 2018	Yes	0	0	0	0	RCT	High
Foerster 2019	No	+	0		+	Cohort study	Moderate
Lange 2015	No		+/0			Cross-sectional	Moderate
Mazzer 2018	No			+		Cohort study	Moderate
Ogunleye 2015	No	+				Cross-sectional	Moderate
Ononogbu 2014	No		+			Cross-sectional	Moderate
Parent 2016	No		+	0		Cross-sectional	Moderate
Perrault 2019	Yes	+		+		Quasi-experimental	Moderate
Twenge 2017	No			+		Cross-sectional	Moderate
Van der Schuur 2018	No		0		0	Cohort study	Moderate
Vandendriessche 2019	No	+		+		Cross-sectional	Moderate
**Electronic media devices in the bedroom**							
Calamaro 2009				0		Cross-sectional	Moderate
Continente 2016				+/0		Cross-sectional	Moderate

Note: 1st author and year. (+) Significant positive association; (0) No association; (−) Significant negative association; (+/0) refers to both a positive association and no association in different subgroups; (+/−) refers to both a positive association and a negative association in different subgroups

#### Bedtime and sleep onset

Eleven studies investigated the relationship between electronic media use and delayed bedtime and sleep onset [23, 24, 40, 43, 45, 46, 48–51, 59]. Nine of these studies showed a positive association [23, 40, 43, 45, 46, 48, 49, 51, 59]. High electronic media use was associated with problems falling asleep/later sleep onset [23, 40, 45, 46, 51, 59], delayed bedtime [43, 46, 49] and bedtime problems [45] The study by Poulain et al. (2019) showed a positive association for high use of computer or Internet (3–4 h/day or more) and more bedtime problems at 12-month follow-up, while no association was found for television viewing or mobile phone use [45]. Two studies found an association between social media use and delayed sleep onset [46, 48]. Van der Schuur et al. (2019) found that social media stress was longitudinally related to sleep onset latency among girls, but not boys [48]. Scott et al. (2019) found a dose-response relationship between social media use and late sleep onset, where a higher use of social media was associated with higher odds of late sleep onset [46].

#### Sleep quality

Nine studies assessed the association between electronic media use and sleep quality, including restless sleep, night-time awakenings, and insomnia complaints [15, 40, 41, 44–46, 50, 54, 55] and seven of these studies found a positive association [15, 40, 41, 44, 46, 54, 55]. Three of these studies examined social media use [46, 54, 55]. There were indications that a large amount of time on social media or problematic use of social media had an impact on sleep quality [46, 54, 55]. Problematic use of social media was measured by whether the adolescents preferred spending time on social media rather than engaging in social activities or used social media to feel good about themselves.

#### Sleep duration

The relationship between electronic media use and sleep duration was examined in 13 studies [15, 22–26, 38, 39, 42, 50, 51, 58, 59]. Mazzer et al. (2018), who examined eighth- and ninth-grade students over a year, found that of electronic media use was associated with short sleep duration [42]. Regarding different types of electronic media, it appears that computers [22, 24, 38, 58], mobile phones [22, 24, 38], and video games [22, 38] affected sleep duration. Brunetti et al. (2016), for example, found that computer use doubled the odds of a short night’s sleep, while talking on a mobile phone tripled the odds of a short night’s sleep [22]. In contrast, Tavernier et al. (2017) found that talking on the phone increased the sleep duration, whereas texting reduced the sleep duration. In this study, social media use was not related to sleep duration, whereas Twenge et al. (2017) found an association between social media and short sleep duration. Two studies investigated the presence of electronic media in the bedroom and sleep duration. There were indications that a computer, but not a television or gaming console, in the bedroom negatively affected sleep duration [23, 39].

#### Daytime tiredness

Eight studies examined the association between electronic media use and daytime tiredness, and found mixed results [22, 23, 40, 45, 47–50]. Poulain et al. (2019) showed that high computer or Internet use, but not television and mobile phone use, resulted in more daytime tiredness [45]. Brunetti et al. (2016) found that computer use and time spent talking on the mobile phone were associated with more daytime sleepiness while no associations were found for videogame time and television use [22]. One study that examined social media showed that using social media was not in itself associated with daytime tiredness, but adverse emotional reactions arising from social media (i.e. social media stress) was related to daytime tiredness among girls [48].

## Discussion

This systematic review summarizes results from 49 epidemiological studies on associations between electronic media use and sleep in 0–15-year-old children and adolescents. Across age groups, we found consistent evidence that media use was associated with short sleep duration. The evidence for a relationship between electronic media use and other sleep outcomes was less strong.

For the youngest children (i.e. preschool children), television watching, and tablet device use were associated with difficulties in falling asleep and less sleep duration. Moreover, heavier television use was associated with increased daytime napping, which suggests poorer sleep consolidation and less mature sleep patterns [60]. There was no or insufficient evidence for an association of video game, mobile phone, computer, and the presence of an electronic media device in the bedroom with poor sleep outcomes among 0–5-year-olds.

For 6–12-year-old children, use of electronic media (television, video game console, mobile phone, computer, screen time) in general and at bedtime and their presence in the bedroom was associated with later bedtimes and shorter sleep duration. Additionally, we found evidence for an association of bedtime television and mobile phone use as well as total screen time with sleep disturbances and awakening at night. Similar results have been reported in a previous systematic review examining the association between portable screen-based media device access or use in the bedroom and less sleep [61]. Our results support the hypothesis that evening exposure to bright light from screens may disturb the sleep-wake cycle and suppresses the melatonin production [7, 62]. Other mechanisms through which media use may interfere with sleep onset and sleep problems are time replacement (i.e. time spent on the screens at night displaces time spent sleeping) or the psychological stimulation from the media content [63].

For 13–15-year-olds, there was evidence for a positive association of total screen time and use of computer and mobile phone with less sleep. Moreover, the included studies indicated that screen time was associated with problems falling asleep, and social media use was associated with poor sleep quality. Television watching was least likely to be associated with poor sleep outcomes. Thus, for this age group our study supports that more interactive forms of electronic media with increases in physiological arousal [64] may have greater impact on sleep than more passive forms [7, 65]. Our results are in line with a systematic review [66] including an older age group (15–24-year-olds) that suggests that adolescents often use electronic devices for social media, and this may explain the relationship between use of electronic media device and poor sleep.

Five of the included studies investigated subgroup effect or effect modification, i.e. whether an observed association differed depending on characteristics of the study sample. Four studies performed gender-specific analyses [39, 40, 48, 49] and one study examined ethnicity [12]. The studies found that the association between electronic media use and sleep may depend on gender and ethnicity. For example, a study from US found that bedroom TV was associated with less sleep for ethnic minority children but not among white non-Hispanic children [12]. Future studies of properly powered subgroup analysis should further investigate whether the association between media use and sleep in children and adolescent is differentially impacted by factors such as age and ethnicity [67]. Moreover, how does parenting style, values, and socioeconomic status of the family contribute to the impact of electronic media on sleep. Improvements in sociocultural and contextual understanding would elucidate the association between media use and sleep in childhood and adolescence [68].

### Limitations and future directions

We noted several limitations in the studies included in this review. Firstly, most of the study designs were cross-sectional which precluded causal inferences and limit the conclusions of this review because it is not possible to uncover the direction of relationships between electronic media and sleep. Some children and adolescents may experience bedtime procrastination (i.e. going to bed later than intended despite the absence of external reasons) [69] and use electronic media as an activity before sleep. Others may use electronic media to help them go to sleep [70] or because of tiredness [42]. Such reciprocal associations are confirmed by some of the included studies [42, 45]. Prospective studies with measurements at multiple time points are needed to identify how and when use of electronic media impact on sleep in childhood and youth. Secondly, both self-reported and parent-reported data may be subject to uncertainty. For example, adolescents tend to over-report their sleep duration compared with objective measurements such as actigraphy or diary methods [71], and parents tend to report better sleep for adolescents compared with both self-reported and objective measurements [72]. Thirdly, the included studies were measuring media use and sleep outcomes differently (e.g., overall screen time exposure versus bedtime use). Due to the substantial heterogeneity in measurements of media and sleep as well as in effect size measure, it was difficult to summarize the results, estimate the magnitude of the associations and provide clear conclusions. Nonetheless, we have rigorously outlined the associations between electronic media and each sleep outcome which enables comprehensive results; still, there were several insufficiencies. The studies among 6–12-year-olds lacked measurements of sleepiness during daytime; a factor that may have serious consequences on schoolwork and leisure activities. In the age group of 13–15-year-olds, there was a lack of studies measuring the electronic media use at bedtime or during the night. This is an important area to examine because parental monitoring and parent-set bedtimes decline significantly from early to late years of adolescence [73] which might imply an increase in the use of electronic media devices at bedtime.

This review was limited to include studies published in English and study populations from Western countries. The latter make us able to generalize to Western countries but limits the generalizability of our findings to non-Western countries. The reason for the inclusion criteria was that traditions, values, conditions and environments for sleep practices and attitudes may differ between Western and non-Western countries [74], inhibiting results to inform policy and practice.

Despite these limitations, we note several strengths of this study in addressing the association between electronic media device use and sleep in children and adolescents. First, the studies represented a relatively short period of time which ensured that the definition of electronic media devices remained stable. Secondly, we excluded studies of low quality. Thirdly, we included a broad age group; children and adolescents from 0 to 15. This broadens our understanding of how the use of electronic media devices may impact sleep in different age groups in a childhood development perspective. However, comprehensive equity reviews on e.g. socio-economic background in the study population and mechanisms would provide a more thorough understanding of the associations. Only a few qualitative studies were identified. This leaves a large gap in understanding the complexities of electronic media device use and sleep relationship in children and adolescents.

### Implications for policy and practice

At the policy level, information and more public awareness could be promoted about the potential negative impact on children’s sleep of electronic media, if used excessively and immediately before bedtime. In general, this could include renewed awareness and promotion of appropriate sleep hygiene, but also more attention to the potential adverse effects of the seemingly unavoidable increase in the use of electronic media in the everyday life. At the practice level, professionals and caretakers of children and adolescents should have a heightened awareness on sleep and encourage bedtime routines including calming activities without use of electronic media and remove all electronic media from the bedroom [75]. Given the strong attraction of electronic media on most children, interventions should include both structural measures to guide children’s electronic media habits and individual measures focusing on e.g. information about the potential impact of electronic media devices and how to develop healthy media habits.

## Conclusions

This systematic review of 49 studies found consistent evidence that use of electronic devices is associated with shorter sleep duration in children and adolescents. The association between electronic media use and other sleep outcomes was more inconclusive. Moreover, the evidence for association between electronic media and sleep was stronger for 6–15-years-olds than 0–5-year-olds.

## Supplementary Information

**Additional file 1: Table S1**: Search terms and syntax. **Table S2**: Characteristics and detailed results of included studies among 0–5-year-old children. **Table S3**: Characteristics and detailed results of included studies among 6–12-year-old children. **Table S4**: Characteristics and detailed results of included studies among 13–15-year-old children. **Table S5**: References on excluded studies.

## Data Availability

All data generated or analyzed during this study are included in this article (and its supplementary files).

## Abbreviations

N: Number
RCT: Randomized controlled trial
