# Supplementary material for: Electronic media use and sleep in children and adolescents in western countries: a systematic review
Source: BMC Public Health. 2021 Sep 30;21:1598. doi: 10.1186/s12889-021-11640-9 (PMC8482627; doi:10.1186/s12889-021-11640-9)
Supplement: Supplementary file 1 — Additional file 1: Table S1: Search terms and syntax. Table S2: Characteristics and detailed results of included studies among 0–5-year-old children. Table S3: Characteristics and detailed results of included studies among 6–12-year-old children. Table S4: Characteristics and detailed results of included studies among 13–15-year-old children. Table S5: References on excluded studies. [file 12889_2021_11640_MOESM1_ESM.docx]

**eTable 1: Search terms and syntax**

| **Date** | **Database** | **keywords**  **AND / OR / NOT** | **Hits** |
| --- | --- | --- | --- |
| August 23rd  2019 | Web of science | (("Adolescen*" or "Baby" or "Babies" or "Boy*" or "Child*" or "Girl*" or "Infant*" or "Juvenile*" or "Kid*" or "Minor*" or "Newborn*" or "Preschool child*" or "School child*" or "School-aged child*" or "Teen*" or "Teenager*" or "Toddler*" or "Young adult*" or "Young child*" or "Youth*") AND ("Cell phone*" or "Cell Phone Use*" or "Cellular phone*" or "Computer*" or "Computer game*" or "Digital computer*" or "Digital device*" or "Digital media" or "Digital screen*" or "Electronic device*" or "Electronic media" or "Hybrid computer*" or "Ipad*" or "Media device*" or "Mobile phone*" or "Portable media" or "Portable mobile*" or "Screen*" or "Short message service*" or "Smartphone*" or "Social media" or "Tablet computer*" or "Tablet device*" or "Telephone*" or "Television*" or "Text messaging" or "Touch screen device*" or "Video game*" or "Screen exposure*" or "Screen time*" or "Television exposure*" or "Screen based activit*" or "Screen based light*" or "Screen-stacking*" or "Blue light*" or "Light exposure*" or "television viewing") AND ("Bedroom light exposure*" or "Bedtime*" or "Bedtime dela*" or "Bedtime media device use*" or "Bedtime routine*" or "Bedtime screen habit*" or "Bedtime TV-viewing*" or "Sleep initiation*" or "Sleep latenc*” or “Deep sleep” or “Night sleep” or “REM sleep” or “Sleep” or “Sleep cycle*” or “Sleep deprivation*” or “Sleep disturbance*” or “Sleep efficienc*” or “Sleep fragmentation*” or “Sleep habit*” or “Sleep hygiene*” or “Sleep interruption*” or “Sleep maintenance*” or “Sleep pattern*” or “Sleep rhythm*” or “Sleep walking cycle*” or "Daytime function*" or "Daytime sleepiness" or "Daytime tiredness" or "Reduced sleep duration*" or "Shortened sleep duration*" or "Sleep duration*" or "Sleep evaluation*" or "Sleep impact" or "Sleep outcome*" or "Sleep quantit*" or "Sleep qualit*" or "Sleep time*" or "Total sleep" or "Total sleep time*")) | 2,831 |
| August 23rd  2019 | Medline | ((“exp Child/” or “exp Infant, Newborn/” or “exp Infant/” or “exp Child, Preschool/” or “exp Minors/” or “exp Adolescent/” or “exp Young Adult/” or "Adolescen*" or "Baby" or "Babies" or "Boy*" or "Child*" or "Girl*" or "Infant*" or "Juvenile*" or "Kid*" or "Minor*" or "Newborn*" or "Preschool child*" or "School child*" or "School-aged child*" or "Teen*" or "Teenager*" or "Toddler*" or "Young adult*" or "Young child*" or "Youth*") AND (“exp Video Games/” or “exp Smartphone/” or “exp Cell Phone/” or “exp Text Messaging/” or “exp Television/” or “exp Telephone/” or “exp Computers/” or “exp Social Media/” or “exp Computers, Handheld/” or “exp Screen Time/” or “exp Computers, Hybrid/” or " Cell phone*" or "Cell Phone Use*" or "Cellular phone*" or "Computer*" or "Computer game*" or "Digital computer*" or "Digital device*" or "Digital media" or "Digital screen*" or "Electronic device*" or "Electronic media" or "Hybrid computer*" or "Ipad*" or "Media device*" or "Mobile phone*" or "Portable media" or "Portable mobile*" or "Screen*" or "Short message service*" or "Smartphone*" or "Social media" or "Tablet computer*" or "Tablet device*" or "Telephone*" or "Television*" or "Text messaging" or "Touch screen device*" or "Video game*" or "Screen exposure*" or "Screen time*" or "Television exposure*" or "Screen based activit*" or "Screen based light*" or "Screen-stacking*" or "Blue light*" or "Light exposure*" or "television viewing") AND (“exp Sleep Latency/” or “ exp Sleep/” or “exp Sleep Deprivation/” or “exp Sleep Hygiene/” or “exp Sleep, REM/” or "Bedroom light exposure*" or "Bedtime*" or "Bedtime dela*" or "Bedtime media device use*" or "Bedtime routine*" or "Bedtime screen habit*" or "Bedtime TV-viewing*" or "Sleep initiation*" or "Sleep laten*" or “Deep sleep” or “Night sleep” or “REM sleep” or “Sleep” or “Sleep cycle*” or “Sleep deprivation*” or “Sleep disturbance*” or “Sleep efficienc*” or “Sleep fragmentation*” or “Sleep habit*” or “Sleep hygiene*” or “Sleep interruption*” or “Sleep maintenance*” or “Sleep pattern*” or “Sleep rhythm*” or “Sleep walking cycle*” or "Daytime function*" or "Daytime sleepiness" or "Daytime tiredness" or "Reduced sleep duration*" or "Shortened sleep duration*" or "Sleep duration*" or "Sleep evaluation*" or "Sleep impact" or "Sleep outcome*" or "Sleep quantit*" or "Sleep qualit*" or "Sleep time*" or "Total sleep" or "Total sleep time*")) | 3,759 |
| August 23rd  2019 | Embase | ((“exp juvenile/” or ”exp child/” or “exp infant/” or “exp baby/” or ”exp newborn/” or “exp preschool child/” or “exp school child/” or “exp toddler/” or “exp boy/” or “exp girl/” or “exp adolescent/” or “exp minor (person)"/ or “exp young adult/” or “Adolescen*" or "Baby" or "Babies" or "Boy*" or "Child*" or "Girl*" or "Infant*" or "Juvenile*" or "Kid*" or "Minor*" or "Newborn*" or "Preschool child*" or "School child*" or "School-aged child*" or "Teen*" or "Teenager*" or "Toddler*" or "Young adult*" or "Young child*" or "Youth*") AND (“ exp Video game/” or “exp electronic device/” or “exp television/” or “exp television viewing/” or “exp mobile phone/” or “exp smartphone/” or “exp social media/” or “exp tablet computer/” or “exp computer/” or “exp digital computer/” or “exp hybrid computer/” or text messaging/” or “exp light exposure/” or “exp blue light/” or "Cell phone*" or "Cell Phone Use*" or "Cellular phone*" or "Computer*" or "Computer game*" or "Digital computer*" or "Digital device*" or "Digital media" or "Digital screen*" or "Electronic device*" or "Electronic media" or "Hybrid computer*" or "Ipad*" or "Media device*" or "Mobile phone*" or "Portable media" or "Portable mobile*" or "Screen*" or "Short message service*" or "Smartphone*" or "Social media" or "Tablet computer*" or "Tablet device*" or "Telephone*" or "Television*" or "Text messaging" or "Touch screen device*" or "Video game*" or "Screen exposure*" or "Screen time*" or "Television exposure*" or "Screen based activit*" or "Screen based light*" or "Screen-stacking*" or "Blue light*" or "Light exposure*" or "television viewing") AND (“exp sleep/” or “exp sleep latency/” or “exp night sleep/” or “exp sleep pattern/” or “exp sleep quality/” or “exp sleep waking cycle/” or “exp sleep hygiene/” or “exp sleep deprivation/” or “exp sleep time/” or "Bedroom light exposure*" or "Bedtime*" or "Bedtime dela*" or "Bedtime media device use*" or "Bedtime routine*" or "Bedtime screen habit*" or "Bedtime TV-viewing*" or "Sleep initiation*" or "Sleep laten*" or “Deep sleep” or “Night sleep” or “REM sleep” or “Sleep” or “Sleep cycle*” or “Sleep deprivation*” or “Sleep disturbance*” or “Sleep efficienc*” or “Sleep fragmentation*” or “Sleep habit*” or “Sleep hygiene*” or “Sleep interruption*” or “Sleep maintenance*” or “Sleep pattern*” or “Sleep rhythm*” or “Sleep walking cycle*” or "Daytime function*" or "Daytime sleepiness" or "Daytime tiredness" or "Reduced sleep duration*" or "Shortened sleep duration*" or "Sleep duration*" or "Sleep evaluation*" or "Sleep impact" or "Sleep outcome*" or "Sleep quantit*" or "Sleep qualit*" or "Sleep time*" or "Total sleep" or "Total sleep time*")) | 8,211 |
| August 23rd  2019 | CINAHL | ((“MH “Young Adult”” or ”MH “Child, Preschool”” or “MH “Minors (Legal)”” or “MH “Infant, Newborn+”” or ”MH “Adolescence+”” or “MH “Infant+”” or “MH “Child+” or “ Adolescen*" or "Baby" or "Babies" or "Boy*" or "Child*" or "Girl*" or "Infant*" or "Juvenile*" or "Kid*" or "Minor*" or "Newborn*" or "Preschool child*" or "School child*" or "School-aged child*" or "Teen*" or "Teenager*" or "Toddler*" or "Young adult*" or "Young child*" or "Youth*") AND (“MH “Video Games+”“ or “MH “Television”” or “MH “Computers, Portable+”” or “MH “Computers, Hand-Held+”” or “MH “Smartphone”” or “MH “Telephone+”” or “MH “Cellular Phone+”” or “MH “Text Messaging+”” or “MH “Social Media+”” or “MH “Screen Time”” or "Cell phone*" or "Cell Phone Use*" or "Cellular phone*" or "Computer*" or "Computer game*" or "Digital computer*" or "Digital device*" or "Digital media" or "Digital screen*" or "Electronic device*" or "Electronic media" or "Hybrid computer*" or "Ipad*" or "Media device*" or "Mobile phone*" or "Portable media" or "Portable mobile*" or "Screen*" or "Short message service*" or "Smartphone*" or "Social media" or "Tablet computer*" or "Tablet device*" or "Telephone*" or "Television*" or "Text messaging" or "Touch screen device*" or "Video game*" or "Screen exposure*" or "Screen time*" or "Television exposure*" or "Screen based activit*" or "Screen based light*" or "Screen-stacking*" or "Blue light*" or "Light exposure*" or "television viewing") AND (“MH “Sleep Latency”” or “MH “Sleep+”” or “MH “Deep Sleep”” or “MH “Sleep, REM”” or “MH “Sleep Hygiene”” or “MH “Sleep Deprivation”” or "Bedroom light exposure*" or "Bedtime*" or "Bedtime dela*" or "Bedtime media device use*" or "Bedtime routine*" or "Bedtime screen habit*" or "Bedtime TV-viewing*" or "Sleep initiation*" or "Sleep laten*" or “Deep sleep” or “Night sleep” or “REM sleep” or “Sleep” or “Sleep cycle*” or “Sleep deprivation*” or “Sleep disturbance*” or “Sleep efficienc*” or “Sleep fragmentation*” or “Sleep habit*” or “Sleep hygiene*” or “Sleep interruption*” or “Sleep maintenance*” or “Sleep pattern*” or “Sleep rhythm*” or “Sleep walking cycle*” or "Daytime function*" or "Daytime sleepiness" or "Daytime tiredness" or "Reduced sleep duration*" or "Shortened sleep duration*" or "Sleep duration*" or "Sleep evaluation*" or "Sleep impact" or "Sleep outcome*" or "Sleep quantit*" or "Sleep qualit*" or "Sleep time*" or "Total sleep" or "Total sleep time*")) | 1,739 |

**eTable 2: Characteristics and detailed results of included studies among 0-5-year-old children**

| **First author, year, country** | **Sample size and age range (N)** | **Study design** | **Exposure** | **Outcome** | **Results** |
| --- | --- | --- | --- | --- | --- |
| Beyens 2019 US | 3-5 years old  (mean age 4 years). N = 402 | Cross-sectional study  Parent-reported questionnaire data | Overall and evening television use  Overall and evening tablet use  Overall and evening smartphone use  Overall/evening use of handheld game player; computer; video | Bedtime  Wake time  Sleep consolidation  Naptime  Total sleep  Bedtime  Wake time  Sleep consolidation  Naptime and Total sleep  Bedtime, Wake time or Total sleep  Sleep consolidation  Naptime  Bedtime, Wake time, Total sleep, Sleep consolidation, Naptime | β = 0.18, p <.001 and β =0.23, p <.001  β = 0.18, p <0 .001 and β = 0.17, p <0 .01  β = –0.23, p <0 .01 and NS  β =0.25, p < 0.01 and NS  NS  β= 0.30, p <0 .001 and β =0.28, p <.001  β = 0.21, p <0 .001 and β = 0.20, p <0 .001  NS and β = –0.16, p <0.05  NS  NS  β = –0.17, p <0 .05 and β = –0.20, p <0 .01  NS and β =0.17, p < 0.05  NS |
| Cespedes 2014  US | 6 months – 7 years old  N = 1,864 | Prospective cohort study (7 years follow-up)  Parent-reported questionnaire data | Average daily TV viewing (at 6 months and annually from 1–7 years) and   The presence of a bedroom TV (annually 4–7 years). | Sleep duration | *Cross-sectional models*: Greater TV viewing was associated with shorter sleep duration.  *Longitudinal models:*  Each additional hour per day of lifetime TV viewing was associated with 7 fewer minutes per day of sleep (95% CI: 4 to 10).  For ethnic minority children, bedroom TV from 4-7 years was associated with -32 min/day sleep (95% CI: 18 to 46) |
| Cheung 2017  UK | 6 - 36 months  (mean age 19,5 months)  N =715 | Cross-sectional study  Parent-reported questionnaire data | Daily exposure to TV  Use of touchscreens | Sleep onset Frequencies of night awakenings Night-time sleep duration  Daytime sleep duration  Total amount of sleep  Sleep onset  Frequencies of night awakenings  Night-time sleep duration  Daytime sleep duration  Total amount of sleep | NS NS  NS β=-0.093, p<0.02  NS  β=0.213, p<0.001  NS  β=-0.291, p<001  β=0.139, p<0.05  β=-0.146, p<0.01 |
| Garrison et al. 2012  US | 3-5 years old (mean age 51,2 months)  N=565 | Randomized controlled trial. Follow-up after 6, 12 and 18 months. Parent-reported data. | Encouraging parents to replace violent or age inappropriate content with instructive and prosocial content on digital devices; focus on television and film | Sleep problems | OR=0.36; 95% CI: 0.16 to 0.83, p=0.02 |
| Marinelli 2014  Spain | 2-9 years old  N =1,713 | Prospective cohort study  Two cohorts assessed children at age 2 and 4 One cohort assessed children at age 6 and 9.  Parent-reported questionnaire | Increment in TV viewing duration (from less than 1.5 hours per day to 1.5 hours or more per day)  Increment in TV viewing duration during weekends | Total sleep duration  Total sleep duration | β = −0.21, p<0,05.  β = -0.19, p<0.05 |
| McDonald et al. 2014  England | 14-27 months (mean 15.8)  N= 1,702 | Cross-sectional study  Parent-reported questionnaire | >1 h of TV viewing in the morning  >1 h of TV viewing in the evening | Night-time sleep duration < 11 hours   Night-time sleep duration < 11 hours | NS  OR=1.89, 95% CI: 1.26-2.84, p<0.05 |
| Moorman and Harrison 2019  US | 4-5 years old (mean age 56 months)  N=278 | Cross-sectional study  Parent-reported questionnaire data  Outcomes:  Bedtimes weekday  Bedtimes weekend  Wake-up times weekday  Wake-up times weekend Daily nap  Nightly sleep  Only statistically significant associations are shown in results | TV use weekday morning  TV use at daycare  TV use after daycare  TV use weekday at bedtime  TV use weekend morning  TV use weekend at bedtime  DVD/VHS use weekday morning; at daycare; after daycare  DVD/VHS use weekday at bedtime  DVD/VHS use weekend morning  DVD/VHS use weekend midday  DVD/VHS use weekend at bedtime  Gaming weekday morning; after day care  Gaming weekday at daycare  Gaming weekday before bedtime  Gaming weekend morning  Gaming weekend midday  Gaming weekend before bedtime  Internet use weekday morning  Internet use at other times  Presence of TV in bedroom  Presence of DVD/VCRs, video games or computers in bedroom  Sneaky media use (i.e., using a screen media when supposed to be sleeping) | Wake-up times weekday  Nightly duration of sleep   Wake-up times weekend Daily duration of nap  Bed- and wake-up time, sleep duration  Bedtimes weekday  Wake-up times weekday  Nightly duration of sleep  Bedtimes weekend  Wake-up times weekday  Wake-up times weekend  Bed- and wake-up time, sleep duration  Bed- and wake-up time, sleep duration  Wake-up times weekday  Daily duration of nap  Bed- and wake-up time, sleep duration  Daily duration of nap  Bed- and wake-up time, sleep duration  Bedtimes weekend  Wake-up times weekday  Bedtimes weekend  Bedtimes weekend  Wake-up times weekend  Bedtimes weekend  Wake-up times weekend  Bed- and wake-up time, sleep duration  Bedtimes weekday  Bedtimes weekend  Bed- and wake-up time, sleep duration  Bedtimes weekday  Bed- and wake-up time, sleep duration  Bedtimes weekday  Bedtimes weekend  Wake-up times weekday  Wake-up times weekend Daily nap  Nightly sleep | β = 0.18, p<0.05  β = 0.22, p<0.01  β = 0.27, p<0.001  β = 0.18, p<0.05  NS  β = 0.29, p<0.01  β = 0.21, p<0.05  β = 0.26, p<0.05  β = -0.17, p<0.05  β = -0.26, p<0.01  β = -0.26, p<0.01  NS  NS  β = 0.18, p<0.05  β = 0.21, p<0.05  NS  β = 0.21, p<0.05  NS  β = -0.22, p<0.05  β = -0.22, p<0.05  β = 0.27, p<0.01  β = -0.25, p<0.001 β = -0.21, p<0.05  β = -0.29, p<0.01 β = -0.19, p<0.01  NS  β = 0.22, p<0.05  β = 0.19, p<0.05  NS  β = 0.20, p<0.05 NS  β =0.28, p<0.001 β =0.45, p<0.001  β =0.15, p<0.05  β =0.23, p<0.001  β =0.18, p<0.01  β = −0.39, p<0.001 |
| Nathanson and Beyens 2018 US | 3-5 years old  N=402 | Cross-sectional study  Parent-reported questionnaire data | Overall tablet use  Overall smartphone use, gameplayer use, or laptop use  Evening tablet use  Evening smartphone use  Evening gameplayer use or laptop use  Overall television viewing  Evening television viewing | Sleep duration  Bedtime resistance  Daytime sleepiness  Sleep duration, Bedtime resistance or Daytime sleepiness  Sleep duration  Bedtime resistance  Daytime sleepiness  Bedtime resistance  Sleep duration or Daytime sleepiness  Sleep duration, Bedtime resistance or Daytime sleepiness  Sleep duration  Bedtime resistance  Daytime sleepiness  Sleep duration  Bedtime resistance  Daytime sleepiness | β = 0.13, p<0.05  β = 0.17, p<0.01  NS  NS  β = 0.012, p<0.05  β = 0.017, p<0.01  NS  β = 0.011, p<0.05  NS  NS  β = 0.17, p<0.001  β = 0.13, p<0.05  β = 0.18, p<0.001  β = 0.15, p<0.01  β = 0.15, p<0.01  β = 0.19, p<0.001 |
| Parent et al. 2016 US | 3-7 years old  N = 209 | Cross-sectional study  Parent-reported questionnaire data | Weekly screen time | Sleep disturbances  Sleep duration | β= 0.17, p<0.05  β =0.29, p<0.05 |
| Plancoulaine et al.  2018  France | 2, 3 and 5-6 years old  N=1,205 | Prospective cohort study, data collected at age 2, 3 and 5-6 years  Parent-reported questionnaire data | Hours per day spent in watching television or other screens. | Short sleep duration  Medium or long sleep duration  Change from long sleep duration to medium sleep duration | OR = 2.11, 95% CI: 1.50 to 2.97, p<0.01  NS  OR = 0.57, 95% CI: 0.35 to 0.93, p<0.05 |
| Ribner and McHang 2019  US, England, Netherlands | Under 6 months (mean age 4.3 months)  N=429 | Cross-sectional study  Parent-reported questionnaire data | Screen time | Total sleep  Night sleep  Day sleep | β =-0.18, p<0.05  β =-0.21, p<0.001  NS |
| Xu et al. 2016  Australia | 2, 3.5 and 5 years old  N=497 | Prospective cohort study  Face-to-face interviews with parents | One hour/day increase in screen time | Noctunal sleep duaration (hours)  Bedtime (hours)  Sleep latency (minutes)  Wake at night  Sleep duration ≥10 hours | β = -0.05, 95% CI: -0.009 to –0.01, p<0.05  β = 0.07, 95% CI: 0.03 to 0.10, p<0.0001  β = 1.58, 95% CI: 0.53 to 2.63, p<0.01  OR = 1.53, 95% CI: 1.10 to 2.14, p<0.05  OR = 0.87, 95% CI: 0.76 to 1.00, p=0.05 |
| Zhang et al. 2019  Australia | 12-28 months  (mean 19.8)  N=173 | Cross-sectional study  Parent-reported questionnaire data | Screen time | Nocturnal sleep duration Nocturnal sleep variability  Sleep problems | NS  NS NS |

**eTable 3: Characteristics and detailed results of included studies among 6-12-year-old children**

| **First author, year, country** | **Sample size and age range** | **Study design** | **Exposure** | **Outcome** | **Results** |
| --- | --- | --- | --- | --- | --- |
| Arora et al. 2014 England | 11-13 years old  N=959 students | Cross-sectional study  Parent-reported questionnaire data | Television viewing before going to bed on weekdays  Video games before going to bed on weekdays  Mobile telephone use before going to bed on weekdays  Using computer/ laptop for studying before going to bed on weekdays  Using Internet for social networking before going to bed on weekdays | Sleep duration (h)  Awakening several times at night  Difficult falling asleep  Sleep duration (h)  Awakening several times at night  Difficult falling asleep  Sleep duration (h)  Awakening several times at night  Difficult falling asleep  Sleep duration (h)  Awakening several times at night  Difficult falling asleep  Sleep duration (h)  Awakening several times at night  Difficult falling asleep | β= -0.34, p<0.01  OR=4.05, 95% CI: 2.06–7.98, p<0.05  NS  β= -0.47. p<0.01  OR=2.72, 95% CI: 1.39–5.34, p<0.05  OR=2.41, 95% CI: 1.26–4.59, p<0.05  β= -0.75, p<0.001  OR=2.92, 95% CI: 1.56–5.47, p<0.05  OR=1.79, 95% CI: 1.02–3.15, p<0.05  β= -0.45, p<0.01  OR= 1.99, 95% CI: 1.02–3.88, p<0.05  NS  β= -0.86, p<0.001  OR= 3.50, 95% CI: 1.91–6.42, p<0.05  OR=2.59, 95% CI: 1.51–4.43, p<0.05 |
| Brambilla et al. 2017 Italy | 1-14 years old (median age 5.3)  N=2,030 | Cross-sectional study  Parent-reported interview data | Has TV in bedroom    Use of display devices | Total sleep duration (h) Optimal sleep condition  Total sleep duration (h) Optimal sleep condition | NS  OR=0.63, 95% CI: 0.50, 0.79, p<0.001  β= -0.25, p<0.001  NS |
| Barlett et al. 2012  USA | 6-12 years old (mean age 9.6)  N=1,317 | Prospective cohort study, 7 months follow-up  Parent-reported questionnaire data | Total screen time (i.e. watching TV, playing video games, and online time) | Sleep duration | Baseline screen time was associated with follow-up screen time (β=0.52, p<0.001), which was associated with sleep at follow-up (β= --0.16, p<0.001) |
| Chahal et al. 2012  Canada | 10-11 years old  N=3,398 | Cross-sectional study  Self-reported and parent-reported questionnaire data | TV, DVD or video game in bedroom  Computer in bedroom  Handheld device in bedroom e.g. mobile phone  Watch TV or movies in night-time  Gaming in night-time  Use of Internet in night-time   Chat online or on social network   Use a phone to chat or text  Number of devices in bedroom   - 1 - 2 - 3   Number of activities using devices at night-time   - 1 or 2 - 3   Using devices most or all nights | Sleep duration | β=-0.08, p<0-05  β=-0.13, p<0.05  β=-0.12, p<0.05  β=-0.07, p<0.05  NS  β=-0.06, p<0.05  β=-0.08, p<0.05  NS  β=-0.08, p<0.05  β=-0.17, p<0.05  β=-0.20, p<0.05  β=-0.07, p<0.05  β=-0.09, p<0.05  β=-0.07, p<0.05 |
| Falbe et al. 2015  USA | 9-13 years old (mean age 10.6 years)  N=2,048 | Cross-sectional study  Self-reported questionnaire data | Presence of small screens in children’s sleep environment  Presence of TV in children’s sleep environment  Screen time watching TV or DVD’s  Screen time using video or computer games | Sleep duration  Perceived insufficient rest or sleep  Sleep duration  Perceived insufficient rest or sleep  Sleep duration  Perceived insufficient rest or sleep  Sleep duration  Perceived Insufficient rest or sleep | β= -20.6, p<0.0001  β=-1.39, p<0.001  β=-18.0, p<0.001  NS  β=-3.6, p<0.001  β=1.05, p<0.001  β=-5.1, p<0.001  β=1.05, p<0.01 |
| Gentile et al. 2014  USA | 9 years old  N=1,323 | Prospective cohort study, during a school year  Self-reported questionnaire data | Parental monitoring total screen time | Sleep duration | β = 0.03, p<0.05 |
| Greever et al. 2017  USA | 7-12 years old (mean 8.4 years)  N=55 | Cross-sectional study  Self-reported and parent-reported questionnaire data | Screen time | Sleep quality  Sleep duration | β = 0.50, p = 0.02  NS |
| Huss et al. 2015  Netherlands | 6.7 - 8.5 years old (mean age 7.4 years)  N=2,361 | Prospective cohort study (baseline age 5, follow-up age 7)  Parent-reported questionnaire data | Use of mobile phone, 3 times per week (age 5) | Sleep onset delay  Sleep duration  Night wakenings  Parasomnias  Daytime sleepiness Bedtime resistance | NS  IRR=1.43, 95% CI: 1.00 to 2.03 IRR=1.51, 95% CI: 1.02 to 2.23  IRR=1.30, 95% CI: 1.04 to 1.63  NS  OR=2.08, 95% CI: 1.41 to 3.06 |
| Mindell et al. 2016 USA | 2-12 years old (mean 6 years)  Intervention N=76  Control  N=76 | Randomized controlled trial. Follow-up after one month.  Parent-reported questionnaire | Information-campaign (“Sleep Well!”) targeting parents (mothers) on childrens sleep:  1) bedtime before 21:00,  (2) avoid caffeine  (3) keep electronics out of the bedroom | Bedtime before 21  Sleep duration (h) | NS  Intervention: baseline=9.75 hours, follow-up= 10.19 hours, p=0.04  Control: NS |
| Mireku et al. 2019  England | 11-12 years old  N=6,616 | Cross-sectional study  Self-reported and parent-reported questionnaire data | Night-time use (within 1 h before sleep) of at least one screen-based media device  Weekday=WD; Weekend=WE  Night-time use of mobile phones  Weekday=WD; Weekend=WE  Night-time use of televisions Weekday=WD; Weekend=WE | Late wake time  Sleep onset latency  Short sleep duration  Abnormal catch-up sleep  Social jetlag    Late wake time Sleep onset latency  Short sleep duration  Abnormal catch-up sleep  Social jetlag  Late wake time Sleep onset latency  Short sleep duration  Abnormal catch-up sleep  Social jetlag | WD: NS; WE: OR=2.39, p<0.05  WD: OR=0.62, p<0.05; WE: NS  WD: OR=1.81, p<0.05; WE: OR=1.39, p<0.05  OR=1.59, p<0.05  OR=1.89, p<0.05  WD: NS; WE: OR=2.22, p<0.05  WD: NS; WE: NS  WD: OR=1.88, p<0.05; WE: OR=1.56, p<0.05  OR=1.46, p<0.05  OR=1.90, p<0.05  WD: OR=2.38, p<0.05; WE: OR=1.65, p<0.05  WD: OR=0.67, p<0.05; WE: NS  WD: OR=1.45, p<0.05; WE: NS  NS  NS  The magnitude of the associations was larger if mobile phone or television was used in darkness |
| Nuutinen et al. 2013  Finland | 10-11 years old at baseline  N=353 | Prospective cohort study,  18 months follow-up  Self-reported questionnaire data | Television viewing;  school days or weekend  Using computer;  school days or weekends  Computer in bedroom;  school days or weekends  TV in bedroom;  school days or weekends | Sleep duration  Bedtime  Sleep duration  Bedtime  Sleep duration  Bedtime  Sleep duration  Bedtime | β=-0.09, p<0.05; NS  β=0.10, p<0.01; NS  β=-0.18, p<0.001; NS  β=0.19, p<0.001; β=0.19, p<.001  NS; NS  NS; β=0.25, p<0.05  NS; NS  NS; NS |
| Parent et al. 2016 USA | 8-12 years old  N = 202 | Cross-sectional study  Parent-reported questionnaire data | Weekly screen time | Sleep disturbances  Sleep duration | β = 0.15, p<0.05  β = -0.25, p<0.001 |
| Redmayne et al. 2013 New Zealand | 10-13 years old (mean 12.3 years)  N=373 | Cross-sectional study  Self-reported and parent-reported questionnaires | Using a cordless phone or a mobile phone | Trouble falling asleep  Wake up in the night  Tired at school | NS  NS NS |
| Yland et al. 2015  USA | 9 years old  N= 3,269 | Cross-sectional study  Parent-reported questionnaire data  Self-reported data from interview | >2 h daily screen time watching TV  >2 h daily screen time on computer  >2 h daily screen time gaming  >2 h daily screen time chatting  Parent reported TV use  Parent reported computer use | Sleep duration | β=-0.10, p<0.05  NS  NS  NS  β=-0.05, p<0.001  β=-0.05, p<0.05 |

**eTable 4: Characteristics and detailed results of included studies among 13-15-year-old children**

| **First author, year, country** | **Sample size and age range** | **Study design** | **Exposure** | **Outcome** | **Results** |
| --- | --- | --- | --- | --- | --- |
| Arora et al. 2013 England | 11-18 years old (mean age 13.9 years) N = 632 | Cross-sectional study  Self-reported questionnaire data | Weekday use of:  - Computer  - Mobile telephone  - TV viewing  - Video gaming | Weekday sleep duration (h) | β= -0.39, p<0.01  β= -0.28, p<0.01  β= -0.35, p<0.01  β= -0.39, p<0.01 |
| Bickham et al. 2018  USA | 6^th,^ 7^th^ and 8^th^ grade  N = 529 (total)  Intervention group: N=157  Comparison group: N=379 | Quasi-experimental, waillist study  Posttest immediately after the screen-free event with approximately 7.5 weeks between pre- and posttests.  Self-reported questionnaire data | Take the Challenge program includes 6-week curriculum with: (1) monitoring and analyzing own and peers’ self-reported media use; (2) reading and discussing research on media effects; (3) developing and completing media time-budgeting forms; (4) identifying alternative social and physical activities; 5) creating media communication products to educate others about media literacy.  Followed by a 10-day event “The Challenge” during which students return daily parent signed slips verifying that the student did not use entertainment screen media on the previous day. | Sleep duration (h) | The intervention group increased sleep duration with approximately 10 min while the comparison group reduced sleep duration with 11 min (p= 0.017).  At posttest, the intervention group reported sleeping approximately 25 min more a night than the comparison group. |
| Brunetti et al. 2016 Canada | 14-16 years old (mean age 15.2 years)  N = 1,233 | Cross-sectional study  Self-reported questionnaire data | >2 h/day computer use  >2 h/day TV use  >2 h/day playing videogames  >2 h/day talking on mobile phones  Computer use (h/day)  TV use (h/day)  Videogames (h/day)  Talking on phone (h/day) | Short sleep duration  Long sleep duration  Short sleep duration  Long sleep duration  Short sleep duration  Long sleep duration  Short sleep duration  Long sleep duration  Daytime sleepiness  Daytime sleepiness  Daytime sleepiness  Daytime sleepiness | OR=2.2, 95% CI: 1.4-3.4, p<0.01  OR=0.5, 95% CI: 1.5-6.2, p<0.01  OR=0.5, 95% CI: 0.3-0.8, p<0.01  NS  NS NS  OR=3.0, 95% CI: 1.5-6.2, p<0.01  NS  β= 2.27, p<0.001  NS  NS  β= 3.16, p<0.001 |
| Calamaro et al. 2009  USA | 12-18 years old (mean age 15 years)  N=100 | Cross-sectional study  Self-reported questionnaire data | Multitasking use of digital media between 9 PM to 6 AM  TV in the bedroom | Has difficulty falling asleep  Falling asleep in school  Sleep duration  Sleep duration | p=0.002  p<0.001  p=0.043  NS |
| Continente et al. 2017 Spain | 13-19 years old (mean age 15.9 years)  N = 3,492 | Cross-sectional study  Self-reported questionnaire data | Computer in bedroom  Television in bedroom  Console in bedroom | Total sleep time  Total sleep time  Total sleep time | Boys: Prevalence Ratio=14% (95% CI:1-28%)  Girls: Prevalence Ratio=26% (95% CI:10-44%)  NS   NS |
| Das-Friebel et al. 2018 Switzerland | 7^th^, 8^th^, 9^th^ and 10^th^ grade.  Mean age 15.1 years  N=352 (total)  Intervention: N=192 Control: N=160 | Cluster RCT study  Follow-up after approximately four weeks (range 20-35 days)  Self-reported questionnaire data | 25-minutes psychoeducation during school including information on importance of sleep and three sleep hygiene rules: (1) Avoid less than 8 h sleep per night; (2) Avoid electronic media use during the last hour before sleep and switch off electronic media devices when going to bed; (3) Avoid/restrict caffeine consumption.  Information leaflet was distributed outlining the sleep hygiene rules, and parents received the leaflets via post. | Sleep duration  Daytime tiredness  Sleep difficulties | NS  NS  NS |
| Foerster et al. 2019  Switzerland | 10-17 years old, 7th to 9th grade (79% were >13 – ≤15 years)  N=895 | Prospective cohort study,  1-year follow-up  Self-reported questionnaire data.  In addition, mobile phone use records were  obtained from mobile phone operators if participants gave additional informed consent. | Nocturnal awakenings due to incoming mobile phone calls or text message  High daily screen time  (above baseline median 180.8 min/day and follow-up median 173.6 min/day) | Problems falling asleep  Restless sleep  Nocturnal awakenings  Early morning awakenings  General sleep quality  Tiredness  Exhaustibility  Lack of energy  Lack of concentration  Problems falling asleep  Restless sleep  Nocturnal awakenings  Early morning awakenings  General sleep quality  Tiredness  Exhaustibility  Lack of energy  Lack of concentration | OR=3.44, 95% CI: 1.03–11.54  OR=5.39, 95% CI: 2.13–13.65  NS  NS  NS  NS  NS  NS  NS  OR=2.35, 95% CI: 1.27–4.34  NS  NS  NS  NS  NS  OR=2.23, 95% CI: 1.21–4.13  NS  OR=3.18, 95% CI: 1.56–6.48 |
| Lange et al. 2015 Germany | 11-17 years old (mean age 14.2 years)  N=7,533 | Cross-sectional study  Self-reported questionnaire data | Television 0.5-2 h/day  Television >3 h/day    Video games 0.5-2 h/day  Video games >3h/day  Computer/internet 0.5-2 h/day  Computer/internet >3h/day  Mobile phone 0.5-2 h/day  Mobile phone >3 h/day  Total screen time 4 to <8 h/day  Total screen >8 h/day | Insomnia | NS  NS  Boys: OR=0.60, p<0.05; girls: NS  NS  NS  Boys: OR=2.56, p<0.05; Girls: NS  NS  NS  NS  Boys: OR=2.45, p<0.01; Girls: NS |
| Mazzer et al. 2018 Sweden | 8th and 9th grade  N=1,620 | Prospective cohort study, 1 year  Self-reported questionnaire | Screen time school day (hours) | Sleep duration | -4.8 min, p<0.001 |
| Nuutinen et al. 2014  Finland, France,  Denmark | 15 years old (mean age 15.6 years)  N=5,402 | Cross-sectional  Self-reported questionnaire | Computer use:  - Finland  - France  - Denmark | Sleep duration (h) | -0.25, p<0.01  -0.19, p<0.01  -0.25, p<0.01 |
| Ogunleye et al. 2015 England | 11-15 years old  N=1,332 | Cross-sectional study  Self-reported questionnaire data | Screen time:  >4 h vs. <2 h  2-4 h vs. <2 h | Late bedtime on weekdays | OR=1.70, p<0.05  OR=1.43, p<0.05 |
| Ononogbu et al. 2014  Finland | 10-14 years old  N=61 | Cross-sectional study  24-hour with Holter monitor (sleep) and  activity diary on ICT use | ICT use = Hours spent on mobile phones, computer games, internet surfing  High ICT users (1+ h) vs. Low ICT users (<1 h) | Quality of sleep during a school week (indicated by physiological measures) | High ICT users showed a lower sleep time standard deviation of normal to normal interval (SDNN) measures in comparison to low ICT users, p=0.035 |
| Parent et al. 2016 USA | 13-17 years old  N = 210 | Cross-sectional study  Parent-reported questionnaire data | Screen time | Sleep disturbances  Sleep duration | β= 0.23, p<0.01  NS |
| Poulain et al. 2019  Germany | 10-17 years old (mean age 13 years)  N=467 | Prospective cohort study.  Average follow-up time: 12.4 months  Self-reported questionnaire data | TV/video use  Computer/internet use  Mobile phone use | Bedtime problems  Sleep behavior problems  Daytime sleepiness  Bedtime problems  Sleep behavior problems  Daytime sleepiness  Bedtime problems  Sleep behavior problems  Daytime sleepiness | NS  NS  NS  β= 1.28, p<0.001 NS  β= 0.32, p<0.05  NS  NS  NS |
| Scott et al. 2019  England | 13-15 years old  N=11,872 | Cross-sectional study  Self-reported questionnaire data | Using social media <1 h (low users) vs. 1 to <3 hours  Using social media 3 to <5 h (high users) vs. 1 to <3 hours  Using social media 5+ h (very high users) vs. 1 to <3 hours | Sleep onset school days  Sleep onset free days  Wake times free days  Sleep onset school days  Sleep onset free days  Wake times school days  Sleep onset school days  Sleep onset free days  Wake times school days  Wake times free days  Nighttime awakening | OR=0.61, 95% CI: 0.51 to 0.73, p<0.0001  OR=0.57, 95% CI: 0.49 to 0.68, p<0.001  OR=0.79, 95% CI: 0.67 to 0.93, p<0.01  NS for wake times on school days, sleep onset latency, night awakening  OR=1.23,95% CI: 1.02 to 1.49, p<0.05,  OR=1.31, 9% CI: 1.09 to 1.6, p<0.01  OR=1.56, 95% CI: 1.02 to 2.4, p<0.05  NS for wake times free days, sleep onset latency, nighttime awakening  OR=2.12, 95% 1.83 to 2.5, p<0.001  OR=2.41, 95% CI: 2.08 to 2.79, p<0.001  OR=1.97, 95% CI: 1.32 to 2.93, p<0.01  OR=1.57, 95% CI: 1.32 to 1.87, p<0.001  OR=1.36, 95% CI: 1.1 to 1.66, p<0.01  NS for sleep onset latency |
| Tavernier et al. 2017  USA | 11-18 years old (mean age 14.5 years)  N=71 | Prospective cohort study for 3 consecutive days.  Actigraph monitor for 3 nights (sleep)  Self-reported questionnaire data for three evenings (digital media) | Texting  Talking on the phone  Working on computer  Instant messaging - Using Facebook - Twitter - Playing video games - Watching TV | Sleep latency  Sleep hours  Sleep efficiency  Sleep latency  Sleep hours  Sleep efficiency  Sleep latency  Sleep hours  Sleep efficiency  Sleep latency  Sleep hours  Sleep efficiency | NS  β = -0.24, p<0.05  NS  NS  β = 0.33, p<0.05  NS  NS  β = -0.27, p<0.01  β = -1.32, p<0.05  NS  NS  NS |
| Twenge et al. 2017  USA | 8th, 10th and 12th grade  N=369,595 students  (two samples | Cross-sectional study  Self-reported questionnaire data | Electronic device uses 3+ h/day  Social media every day  Internet news every day  TV viewing 3+ h/day (sample 1)  TV viewing 3+ h/day (sample 2) | Short sleep duration | OR=1.56, 95% CI:1.50-1.62.  OR=1.16, 95% CI:1.11-1.21  OR=1.15, 95% CI:1.10-1.21  OR= 1.06, 95% CI:1.01, 1.11  NS |
| Van der Schuur et al. 2018  Netherlands | 11-15 years old (mean age 12.6 years)  N=1,441 | Prospective cohort study; 3-wave with 3-4- month intervals.  Self-reported questionnaire data | Media multitasking  (Three main media activities: watching TV, sending messages and using social networking sites) | Sleep problems | NS |
| Van der Schuur et al. 2019  Netherlands | 11-15 years old (mean age 12.6 years)  N=1,441 | Prospective cohort study; 3-wave with 3-4- month intervals.  Self-reported questionnaire data | Social media use  Social media stress | Sleep latency  Daytime sleepiness  Sleep latency  Daytime sleepiness | NS  Boys: p < 0.05; Girls: NS  Boys: NS; Girls: p < 0.05  Boys: NS; Girls: p < 0.05 |
| Vandendriessche et al. 2019  Belgium,  Estonia,  Finland,  Germany,  Hungary,  Latvia,  Scotland, Slovenia,  Switzerland, Sweden,  Spain,  Wales | 11-15 years old (mean age 13.7 years)  N=49,403 | Cross-sectional study  Self-reported questionnaire data | Screen time/day (hour) | Sleep duration weekdays (h)  Sleep duration weekends (h)  Sleep onset difficulties | -4.2 min (β = −0.07, 95% CI = −0.07; −0.07),  p< <0.001  -3 min (β = −0.05, 95% CI = −0.05; −0.05),  p< <0.001  β = 0.04, 95% CI = 0.04; 0.05, p< <0.001 |
| Vernon et al. 2015  Australia | 12-18 years old (mean 15 years)  N=1,886 | Cross-sectional study  Self-reported questionnaire data | Problematic social networking use | Sleep disturbance  Sleep quality | β = 0.38, p<0.01  β = -0.10. p<0.01 |
| Vernon et al. 2017  Australia | High school (mean age 14.4 years)  N=874 | Prospective cohort for 3 years.  Self-reported questionnaire data once a year | Problematic social networking use | Sleep disruptions | β = 0.41, p<0.05 |
| Wallenius et al. 2009  Finland | 12-18 years old  N=6,761 | Cross-sectional study  Self-reported questionnaire data | Instrumental motives to gaming  Ritualized motives to gaming | Bedtime  Tiredness  Bedtime  Tiredness | Boys: β = - 0.08, p<0.001; Girls: NS  NS  Boys: β = 0.08, p<0.001; Girls: NS  Boys: β= 0.07, p<0.001; Girls: β=0.08, p<0.001 |

**eTable 5: References on excluded studies**

| **Quantitative studies with low quality assessment:** |
| --- |
| 1. Altan, S. S., et al. (2018). "Factors Affecting Daytime Sleepiness in Adolescents." International Journal of Caring Sciences 11(3): 1840-1848. 2. Bartel, K., et al. (2016). "Protective and risk factors associated with adolescent sleep: findings from Australia, Canada, and The Netherlands." Sleep Medicine 23: 97-103. 3. Bauducco, S. V., et al. (2016). "Sleep duration and patterns in adolescents: correlates and the role of daily stressors." Sleep health 2(3): 211-218. 4. Berchtold, A., et al. (2018). "Daily internet time: towards an evidence-based recommendation?" European journal of public health 28(4): 647-651. 5. Borlase, B. J., et al. (2013). "Effects of school start times and technology use on teenagers' sleep: 1999-2008." Sleep and Biological Rhythms 11(1): 46-54. 6. Bruni, O., et al. (2015). "Technology use and sleep quality in preadolescence and adolescence." Journal of Clinical Sleep Medicine 11(12): 1433-1441. 7. Buxton, O. M., et al. (2015). "Sleep in the modern family: Protective family routines for child and adolescent sleep: Results from the 2014 sleep in America Poll." Sleep 1): A384-A385. 8. Calamaro, C. J., et al. (2012). "Wired at a Young Age: The Effect of Caffeine and Technology on Sleep Duration and Body Mass Index in School-Aged Children." Journal of Pediatric Health Care 26(4): 276-282. 9. Chaput, J. P., et al. (2015). "Associations between sleep patterns and lifestyle behaviors in children: an international comparison." International Journal of Obesity Supplements 5(Suppl 2): S59-65. 10. Chaput, J.-P., et al. (2014). "Electronic screens in children's bedrooms and adiposity, physical activity and sleep: do the number and type of electronic devices matter?" Canadian Journal of Public Health 105(4): e273-279. 11. Chindamo, S., et al. (2019). "Sleep and new media usage in toddlers." European Journal of Pediatrics 178(4): 483-490. 12. Dube, N., et al. (2017). "The use of entertainment and communication technologies before sleep could affect sleep and weight status: a population-based study among children." International Journal of Behavioral Nutrition & Physical Activity 14: 1-15. 13. Fobian, A. D., et al. (2016). "Impact of Media Use on Adolescent Sleep Efficiency." Journal of developmental and behavioral pediatrics : JDBP 37(1): 9-14. 14. Foley, L. S., et al. (2013). "Presleep Activities and Time of Sleep Onset in Children." Pediatrics 131(2): 276-282. 15. Fuller, C., et al. (2017). "Bedtime Use of Technology and Associated Sleep Problems in Children." Lobal Pediatric Health 4: 2333794X17736972. 16. Gamble, A. L., et al. (2014). "Adolescent Sleep Patterns and Night-Time Technology Use: Results of the Australian Broadcasting Corporation's Big Sleep Survey." PLoS ONE 9(11). 17. Garmy, P., et al. (2018). "Insufficient Sleep Is Associated with Obesity and Excessive Screen Time Amongst Ten-Year-Old Children in Sweden." Journal of pediatric nursing 39: e1-e5. 18. Garmy, P., et al. (2012). "Sleep and Television and Computer Habits of Swedish School-Age Children." Journal of School Nursing 28(6): 469-476. 19. Garrison, M. M., et al. (2011). "Media use and child sleep: The impact of content, timing, and environment." Pediatrics 128(1): 29-35. 20. Genuneit, J., et al. (2018). "Media consumption and sleep quality in early childhood: results from the Ulm SPATZ Health Study." Sleep Medicine: 7-10 21. Ghekiere, A., et al. (2019). "Trends in sleeping difficulties among European adolescents: Are these associated with physical inactivity and excessive screen time?" International journal of public health 64(4): 487-498. 22. Gradisar, M., et al. (2011). "The sleep and technology use of americans: Results from the 2011 national sleep foundation's sleep in america poll." Sleep and Biological Rhythms 9 (4): 386. 23. Grover, K., et al. (2016). "Effects of Instant Messaging on School Performance in Adolescents." Journal of Child Neurology 31(7): 850-857. 24. Helm, A. F. and R. M. C. Spencer (2019). "Television use and its effects on sleep in early childhood." Sleep health 5(3): 241-247. 25. Hoedlmoser, K., et al. (2010). "Self-reported sleep patterns, sleep problems, and behavioral problems among school children aged 8-11 years." Somnologie 14(1): 23-31. 26. Johansson, A. E., et al. (2016). "Adolescent Sleep and the Impact of Technology Use Before Sleep on Daytime Function." Journal of pediatric nursing 31(5): 498-504. 27. King, D. L., et al. (2014). "Sleep Interference Effects of Pathological Electronic Media Use during Adolescence." International Journal of Mental Health and Addiction 12(1): 21-35. 28. Lemola, S., et al. (2015). "Adolescents' electronic media use at night, sleep disturbance, and depressive symptoms in the smartphone age." Journal of youth and adolescence 44(2): 405-418. 29. Li, X., et al. (2019). "Sleep mediates the association between adolescent screen time and depressive symptoms." Sleep Medicine 57: 51-60. 30. Magee, C. A., et al. (2014). "Bidirectional relationships between sleep duration and screen time in early childhood." JAMA Pediatrics 168(5): 465-470. 31. Mindell, J. A., et al. (2009). "Developmental aspects of sleep hygiene: Findings from the 2004 National Sleep Foundation Sleep in America Poll." Sleep Medicine 10(7): 771-779. 32. Nogueira, M., et al. (2019). "Addictive video game use: An emerging pediatric problem?" Acta Medica Portuguesa 32(3): 183-188. 33. Paiva, T., et al. (2016). "Mutual relations between sleep deprivation, sleep stealers and risk behaviours in adolescents." Sleep Science 9(1): 7-13. 34. Peracchia, S., et al. (2017). "Longer the game, better the sleep: Intense video game playing is associated to better sleep quality and better daytime functioning." Journal of Cyber Therapy and Rehabilitation 10 (1): 50. 35. Plancoulaine, S., et al. (2015). "Gender-specific factors associated with shorter sleep duration at age 3 years." Journal of Sleep Research 24(6): 610-620. 36. Polos, P. G., et al. (2015). "The impact of Sleep Time-Related Information and Communication Technology (STRICT) on sleep patterns and daytime functioning in American adolescents." Journal of Adolescence 44: 232-244. 37. Przybylski, A. K. (2019). "Digital Screen Time and Pediatric Sleep: Evidence from a Preregistered Cohort Study." Journal of Pediatrics 205: 218-223.e211. 38. Rehbein, F., et al. (2010). "Prevalence and risk factors of video game dependency in adolescence: results of a German nationwide survey." Cyberpsychology, behavior and social networking 13(3): 269-277. 39. Reynolds, A. C., et al. (2019). "Impact of high-frequency email and instant messaging (E/IM) interactions during the hour before bed on self-reported sleep duration and sufficiency in female Australian children and adolescents." Sleep health 5(1): 64-67. 40. Sampasa-Kanyinga, H., et al. (2018). "Use of social media is associated with short sleep duration in a dose-response manner in students aged 11 to 20 years." Acta Paediatrica, International Journal of Paediatrics 107(4): 694-700. 41. Schweizer, A., et al. (2017). "Adolescents with a smartphone sleep less than their peers." European Journal of Pediatrics 176(1): 131-136. 42. Scott, H. and H. C. Woods (2018). "Fear of missing out and sleep: Cognitive behavioural factors in adolescents' nighttime social media use." Journal of Adolescence 68: 61-65. 43. Seguin, D. and V. Klimek (2016). "Just five more minutes please: electronic media use, sleep and behaviour in young children." Early Child Development and Care 186(6): 981-1000. 44. Shimoga, S. V., et al. (2019). "Associations of Social Media Use With Physical Activity and Sleep Adequacy Among Adolescents: Cross-Sectional Survey." Journal of medical Internet research 21(6): e14290. 45. Siomos, K. E., et al. (2010). "Insomnia symptoms among Greek adolescent students with excessive computer use." Hippokratia 14(3): 203-207. 46. Sisson, S. B., et al. (2011). "TVs in the bedrooms of children: does it impact health and behavior?" Preventive Medicine 52(2): 104-108. 47. Sormunen, M., et al. (2016). "Self-reported bedtimes, television-viewing habits and parental restrictions among Finnish schoolchildren (aged 10-11 years, and 2 years later aged 12-13 years): Perspectives for health." European Journal of Communication 31(3): 283-298. 48. Spruyt, K., et al. (2014). "Sleep behavior of underrepresented youth." Journal of Public Health (09431853) 22(2): 111-120. 49. Tambalis, K. D., et al. (2018). "Insufficient sleep duration is associated with dietary habits, screen time, and obesity in children." Journal of Clinical Sleep Medicine 14(10): 1689-1696. 50. Twenge, J. M., et al. (2019). "Associations between screen time and sleep duration are primarily driven by portable electronic devices: evidence from a population-based study of U.S. children ages 0-17." Sleep Medicine 56: 211-218. 51. Vernon, L., et al. (2018). "Mobile Phones in the Bedroom: Trajectories of Sleep Habits and Subsequent Adolescent Psychosocial Development." Child development 89(1): 66-77. 52. Vollmer, C., et al. (2017). "Morningness-eveningness correlates with sleep time, quality, and hygiene in secondary school students: a multilevel analysis." Sleep Medicine 30: 151-159. 53. Woods, H. C. and H. Scott (2016). "#Sleepyteens: Social media use in adolescence is associated with poor sleep quality, anxiety, depression and low self-esteem." Journal of Adolescence 51: 41-49. |
| **Qualitative studies:** |
| 1. Crowder, J. S., et al. (2012). "How did the television get in the child's bedroom? Analysis of family interviews." Preventive Medicine 55(6): 623-628. 2. Godsell, S. and J. White (2019). "Adolescent perceptions of sleep and influences on sleep behaviour: A qualitative study." Journal of Adolescence 73: 18-25. 3. Golem, D., et al. (2019). ""My stuffed animals help me": the importance, barriers, and strategies for adequate sleep behaviors of school-age children and parents." Sleep health 5(2): 152-160. 4. Gruber, R., et al. (2017). "Determinants of sleep behavior in adolescents: A pilot study." Sleep health 3(3): 157-162. 5. Lindsay, A. C., et al. (2018). "Exploring Brazilian immigrant mothers' beliefs, attitudes, and practices related to their preschool-age children's sleep and bedtime routines: A qualitative study conducted in the United States." International Journal of Environmental Research and Public Health 15 (9) (no pagination)(1923). 6. Quante, M., et al. (2019). ""Let's talk about sleep": a qualitative examination of levers for promoting healthy sleep among sleep-deprived vulnerable adolescents." Sleep Medicine 60: 81-88. 7. Smahel, D., et al. (2015). "The impact of digital media on health: children's perspectives." International journal of public health 60(2): 131-137. |
